# Bidirectional Photochemistry of Antarctic Microbial Rhodopsin: Emerging Trend of Ballistic Photoisomerization from the 13-*cis* Resting State

**DOI:** 10.1021/acs.jpclett.2c01974

**Published:** 2022-08-24

**Authors:** Partha Malakar, Ishita Das, Sudeshna Bhattacharya, Andrew Harris, Mordechai Sheves, Leonid S. Brown, Sanford Ruhman

**Affiliations:** † Institute of Chemistry, The Hebrew University of Jerusalem, Jerusalem 9190401, Israel; ‡ Department of Molecular Chemistry and Materials Science, The Weizmann Institute of Science, Rehovot 7610001, Israel; § Department of Physics, University of Guelph, 50 Stone Road East, Guelph, Ontario N1G 2W1, Canada

## Abstract

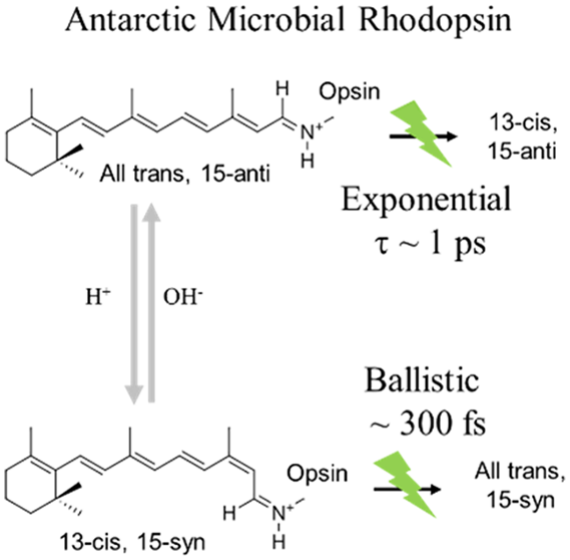

The decades-long ultrafast examination of nearly a dozen microbial retinal proteins, ion pumps, and sensory photoreceptors has not identified structure–function indicators which predict photoisomerization dynamics, whether it will be sub-picosecond and ballistic or drawn out with complex curve-crossing kinetics. Herein, we report the emergence of such an indicator. Using pH control over retinal isomer ratios, photoinduced transient absorption is recorded in an inward proton pumping Antarctic microbial rhodopsin (AntR) for 13-*cis* and *all-trans* retinal resting states. The *all-trans* fluorescent state decays with 1 ps exponential kinetics. In contrast, in 13-*cis* it decays within ∼300 fs accompanied by continuous spectral evolution, indicating ballistic internal conversion. The coherent wave packet nature of 13-*cis* isomerization in AntR matches published results for bacteriorhodopsin (BR) and *Anabaena* sensory rhodopsin (ASR), which also accommodate both *all-trans* and 13-*cis* retinal resting states, marking the emergence of a first structure–photodynamics indicator which holds for all three tested pigments.

The photochemistry of microbial retinal proteins (MRPs) has fascinated photobiologists since their discovery nearly 50 years ago.^1^ Aside from its ultrafast nature, how pigment spectral tuning and/or biological tasks impact photoisomerization dynamics and quantum efficiency and how this process sets the stage for significant energy storage remain subjects of ongoing investigation.^2^ The advent of rapid genome and metagenome sequencing methods has increased the number of known MRPs from a handful two decades ago to thousands, and genes for their expression continue to be identified in DNA of organisms from all domains of life.^3^ Starting with bacteriorhodopsin, ultrafast spectroscopic study of photoisomerization dynamics has now been extended to more than 10 representatives of this protein family.^4−13^ Early expectations that resulting data would uncover significant correlations between pigment absorption wavelengths and/or isomerization rates and/or quantum efficiencies and/or protein function have largely proven unfounded. Such expectations now seem naive in light of the significant sequence variation and range of functionalities within this family of photoreceptors.^14^ Also, while some of the studied pigments are embedded in their natural membrane, others are available only in detergent-stabilized solutions.^15^

It therefore seems that efforts to understand the factors determining reaction dynamics in MRPs may best be initially approached on a protein-by-protein basis. Addressing the diversity in MRP photoisomerization dynamics increasingly involves advanced computational methods.^16−19^ Indeed, maintaining that one “understands” the photochemistry of a specific protein is synonymous with saying it can be reproduced by detailed model calculations with high fidelity. However, if each theoretical model is aimed at reproducing a single reaction it is hard to evaluate their reliability since this process is missing a reference point. For this reason, some of the authors have undertaken the task of characterizing the ultrafast photochemical dynamics in MRPs which can accommodate retinal in more than one resting state.^20,21^ The rationale is that reproducing photoinduced dynamics starting with both resting states in the same protein model presents a much more exacting challenge for assessing the fidelity of a computational model.

The biologically active resting state of most MRPs is *all-trans* with a 15-*anti* Schiff base C=N configuration. Some will, under specific conditions, transform significantly to a double isomerized 13-*cis*, 15-*syn* structure. The first recognized case was BR, which in the dark exists roughly in equal portions of both structures but reverts to *all-trans* over repeated photoexcitation.^22^ A more recent example is ASR, whose name reflects the proposal that photoswitching between these two structures serves for signaling in cyanobacterium *Anabaena*.^23^ In any case, selective photoexcitation considerably alters the abundance of these two resting states in ASR. As a testing ground for computational modeling, the photochemistry of ASR and of BR was recorded with broad-band femtosecond pump–probe spectroscopy under conditions where either resting state is dominant, allowing collection of pump–probe data for each. Interestingly, in both proteins internal conversion from the excited 13-*cis*, 15-*syn* reactant proceeded with continuous spectral evolution and was over within 200 fs, interpreted to result from ballistic coherent curve crossing to S_0_.^20,21^

This similarity in 13-*cis* photochemistry was striking, but defining it as a trend is premature for a sample of just two pigments. The discovery of Antarctic microbial rhodopsin (AntR) presents another candidate for testing photodynamics of numerous resting states in a single opsin. Unlike the former proteins, AntR shifts between the two resting states upon changing pH from an assumed natural alkaline surrounding which overwhelmingly favors an *all-trans*-retinal configuration, to acidic conditions where the 13-*cis* (13CAntR) resting state dominates.^24^ Below we report a first study of ultrafast photochemistry in detergent solutions of AntR differing in pH to accentuate either starting configuration. The results show that *all-trans* AntR (ATAntR) isomerizes with high quantum yield nearly two times slower than BR. In contrast, the 13-*cis*, 15-*syn* reactant state, as in ASR and BR, exhibits ballistic spectral evolution, despite its isomerization with similarly high quantum yield, suggesting that this trait presents a robust predictive structural determinant for ballistic photoisomerization in MRPs.

Transient absorption measurements were carried out on dark-adapted AntR at pH 8, known to contain predominantly *all-trans* retinal. Spectra of the sample and the laser pulses used are shown in Figure 1. Figure 2a shows a color-coded mapping of pump-induced changes in optical density as a function of probe delay and dispersed probe wavelengths along the *y* and *x* axes, respectively. The results have been corrected for the probe continuum chirp, and the plot has a split time axis where the first 1 ps is expanded and presented on a linear scale, while delays from 1 to 100 ps are presented on a logarithmic one. Difference spectra at selected delays are shown in Figure 2b.

**Figure 1:**
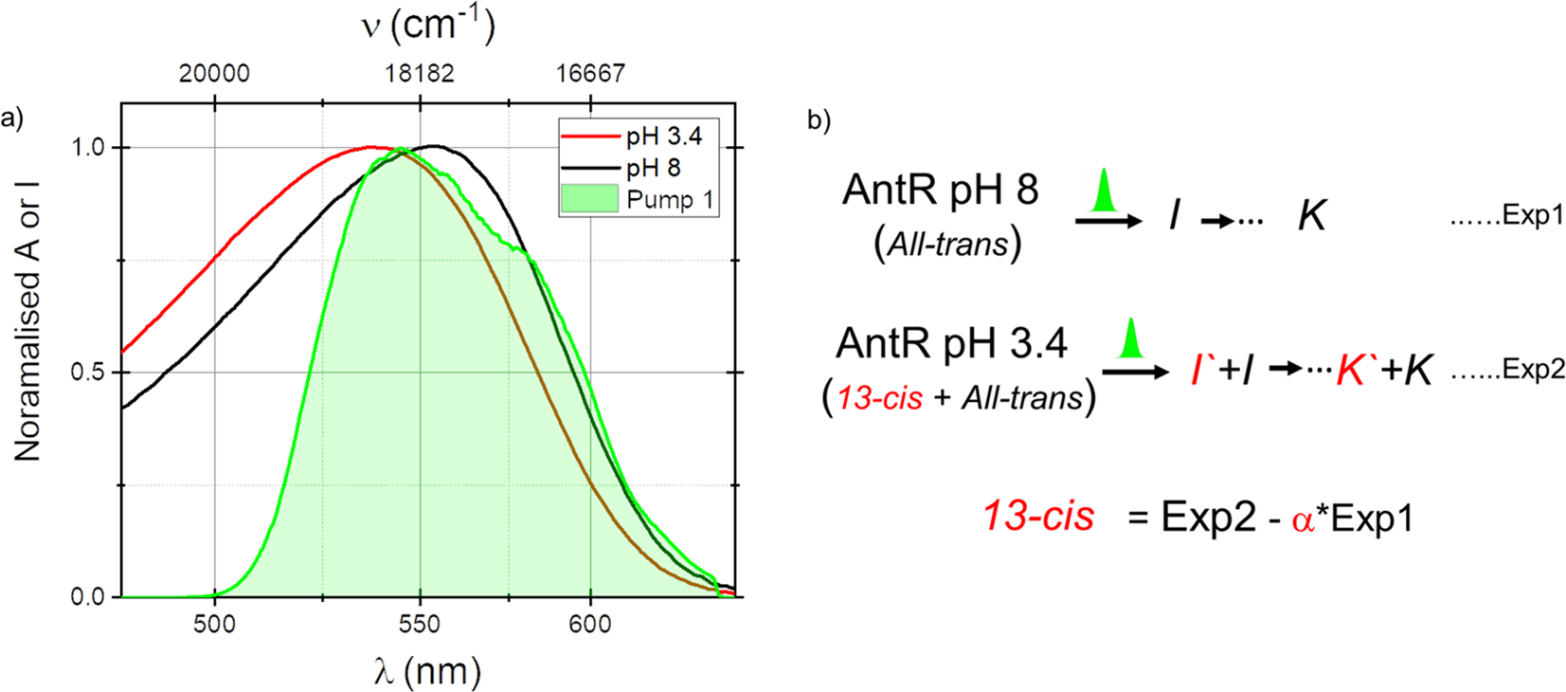
(a) Normalized absorption spectra of dark-adapted AntR at pH 3.4 and pH 8 along with the excitation pulse spectrum in green. (b) A representative scheme of AntR photoreactions at both pH values and isolation of pure 13-*cis* TA data.

**Figure 2:**
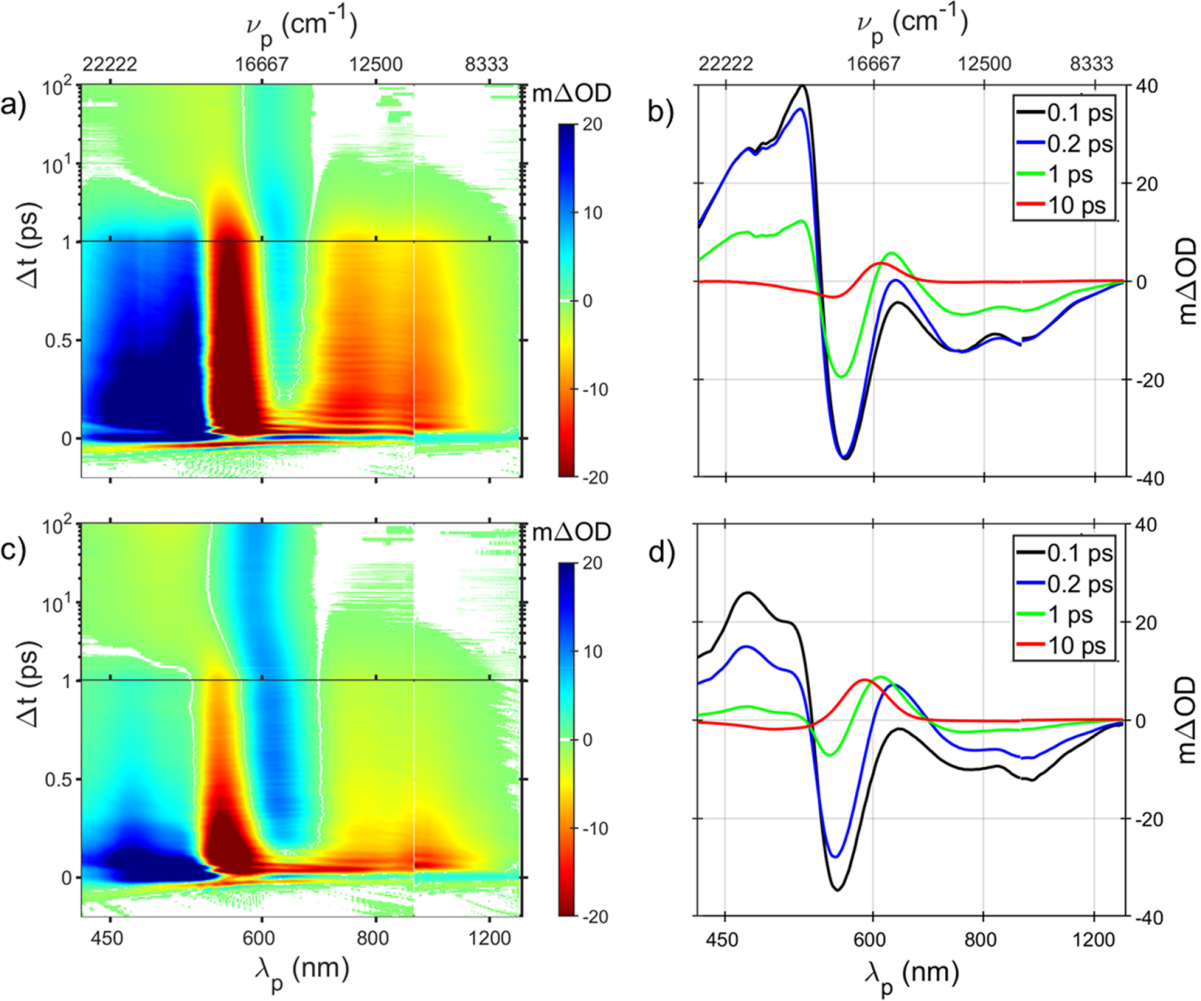
(a) TA data of dark-adapted AntR at pH 8 presented as a 2D color map as a function of probe wavelength (λ_p_)/wavenumber (ν_p_) along *x* and probe delay along *y*. Time axis starts linearly for 1 ps followed by a logarithmic scaling from 1 to 100 ps. ΔOD color-coding is depicted in the attached scale. (b) TA spectra at various pump–probe delays for AntR at pH 8. (c) Same as in panel a for a dark-adapted sample buffered at pH 3.4. (d) As in panel b for pH 3.4.

Following extremely rapid spectral shifts which subside after ∼150 fs, the transient difference spectrum is negative in the range of 540 to 1400 nm. It is composed of an intense band with a sharp minimum at 580 nm due to bleaching of the ground state (GSB) and a broad double-humped negative feature ranging from 600 to 1200 nm. The increase of transmission over this range is attributed to stimulated emission (SE), with a clear dip in transmission assigned to an overlapping excited-state absorption (ESA) band peaking near 800 nm. Below 530 nm an intense ESA with vibronic structure is observed. All these features including rapid spectral shifting over the initial 200 fs concur with previous reports of pump–probe experiments on other MRPs.^4−10^

The fluorescent-state emission decays biexponentially with lifetimes and associated amplitudes of 1 ps (90%) and 5 ps (10%) (Figure S2 and Table S1). The residual difference spectrum with positive and negative maxima at 600 and 565 nm, respectively, is assigned to the superposition of GSB with absorption of a red-shifted metastable ground-state photoproduct. Beyond this delay, the TA spectra remain unchanged for ∼0.1 ns of our measurement and resemble spectra of irradiated ATAntR trapped by cooling to 130 K in the first ground-state photocycle intermediate.^24^ At early delay times, periodic horizontal ripples are observed in the map in Figure 2a. These modulations are assigned to vibrational wave packets in S_1_ and S_0_ due to impulsive excitation. This facet of TA data is described briefly in the Supporting Information and will be fully addressed elsewhere.

A similar experiment conducted on a dark-adapted sample buffered at pH 3.4 is presented in Figure 2c. Under acidic conditions, AntR exists as a mixture of both resting states.^24^ At pH 3, the 13CAntR dominates, shifting absorption of the mixture to 538 nm. Following photoexcitation at pH 3.4, TA spectra show ESA in 430–530 nm ranged and GSB and SE from 530 to 1400 nm. Unlike observations at high pH, a large portion of the excited-state bands decays within a picosecond, accompanied by continuous shifting of the ESA and SE bands as shown in spectra at selected delays in Figure 2d. Later stages of acidic pump–probe data appear like those obtained at pH 8 albeit with lower amplitude and decay further with τ ≈ 1 ps. This suggests that the *all-trans* component of the irradiated sample evolves similarly to that in the alkaline sample, a point essential to the analysis below.

The similarity of the later stages of spectral evolution with that in ATAntR suggests that 13CAntR reacts much faster. The same situation was observed for the 13-*cis* resting states of BR and ASR. To test this, Figure 3 presents finite difference spectra defined as ΔΔOD(ω, *t*, δ*t*) = [ΔOD(ω, *t* + δ*t*) – ΔOD(ω, *t*)], characterizing the change in TA spectra over a specific delay interval δ*t* in both samples. The two sets differ significantly before 0.7 ps, but they resemble one another afterward in ranges where ESA and SE dominate, coinciding once pH 8 data is divided by 2.8 ± 0.2. Henceforth, this is taken as the fractional contributions of ATAntR to the pH 3.4 data.

**Figure 3:**
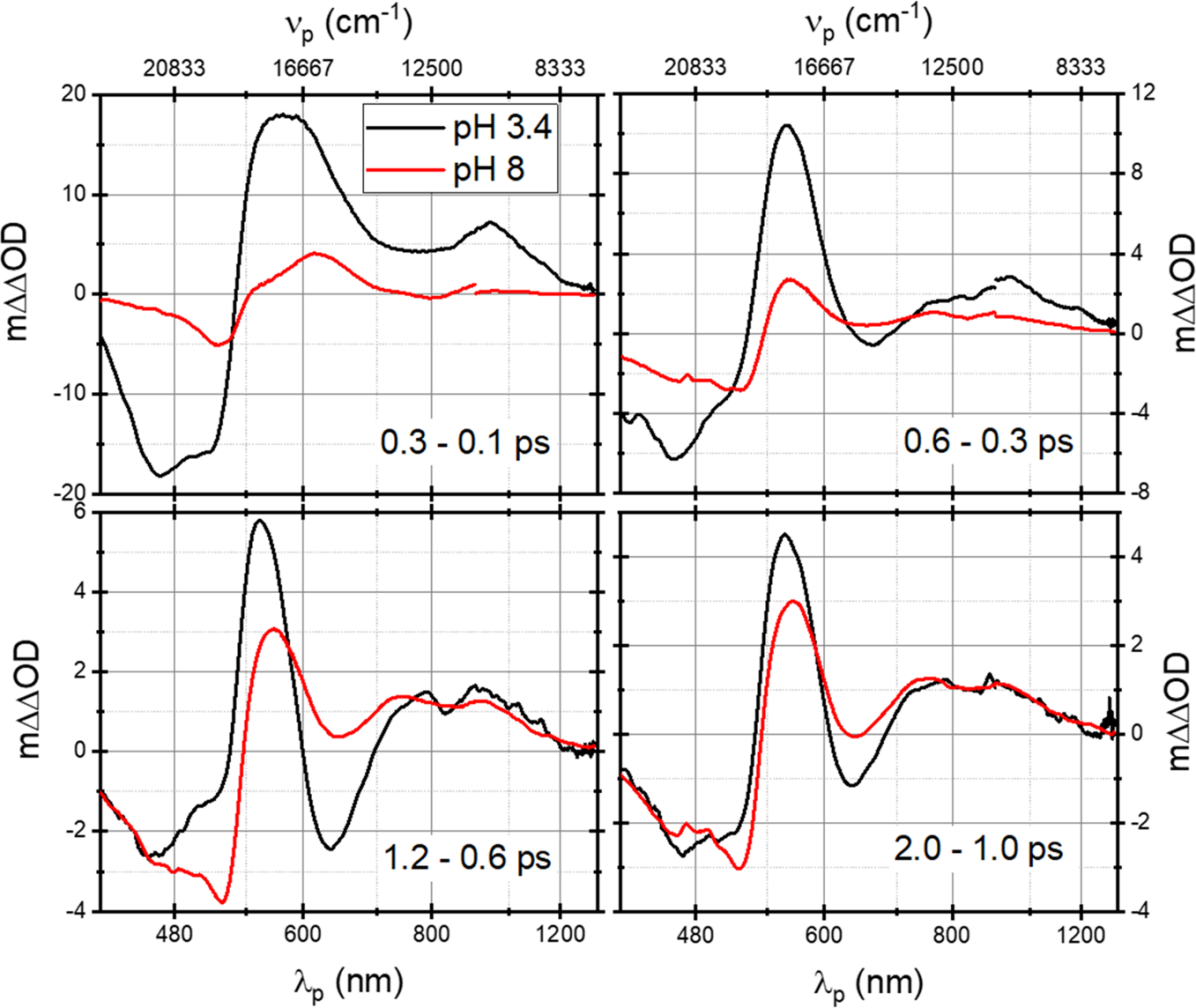
Dynamic difference spectra [ΔOD(*t* + δ*t*) – ΔOD(*t*)], at various stages of excited-state decay. Dynamic difference spectra of pH 8 are divided by 2.8. The relevant delay interval is provided in each graph.

Using this factor, TA data for 13CAntR is extracted by subtraction. Following the coherent artifact during pump–probe overlap, TA spectra presented in Figure 4a show induced absorption above 540 nm that peaks at 500 nm. In addition, the probe range between 540 and 1300 nm is dominated by GSB and SE. Within ∼200 fs, the ESA peak shifts to the blue and diminishes while the emission moves continuously to higher wavelengths and vanishes, both reflecting the continuous evolution toward curve crossing of the 13-*cis* fluorescent state. Similar continuous spectral shifting and rapid disappearance of S_1_ bands was observed for BR and ASR akin to the behavior of bovine rhodopsin simulated computationally in ref (25). Thus, in all the tested MRPs that accommodate both 13-*cis* and *all-trans* resting states, the former consistently exhibits ballistic photochemical dynamics on extremely short time scales.

**Figure 4:**
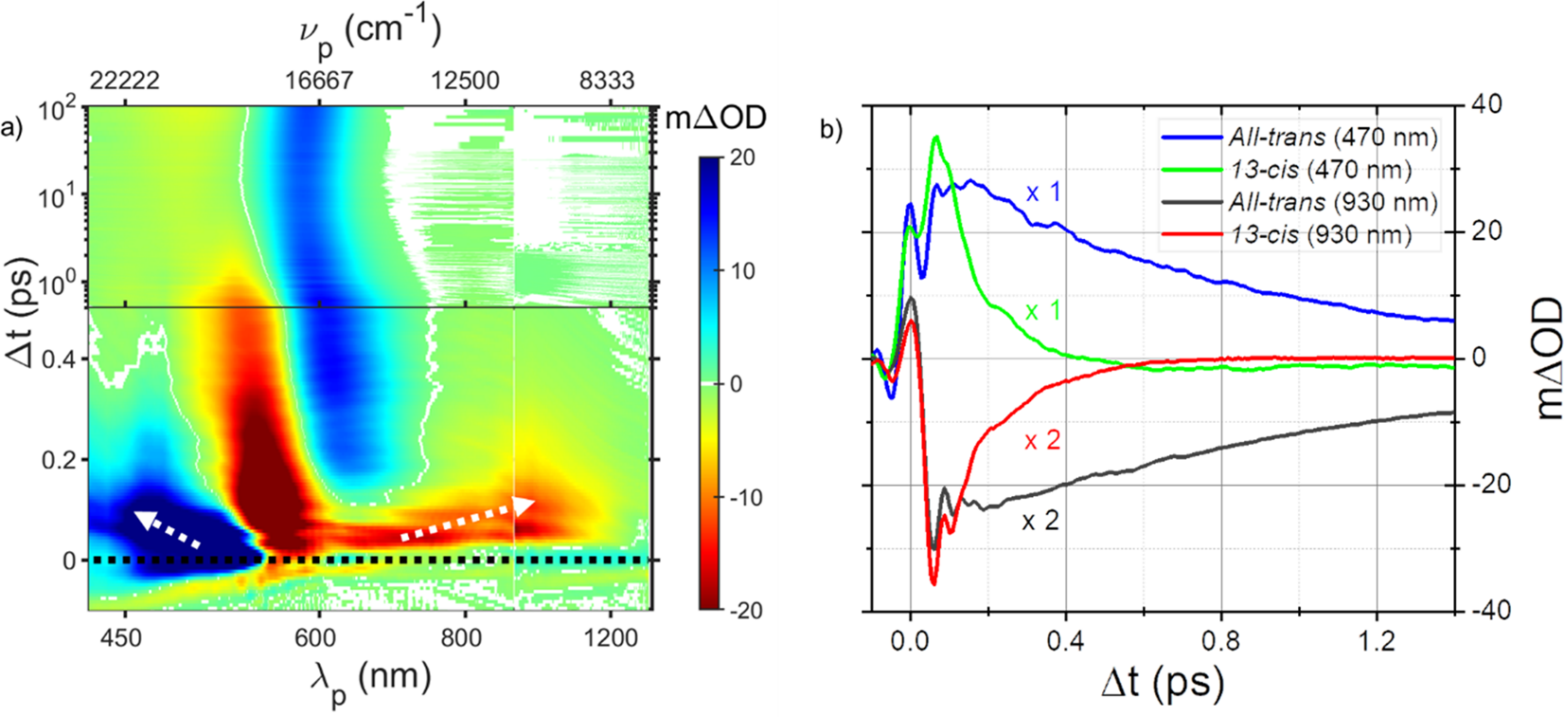
(a) 2D color map of TA data of 13-*cis* AntR. Time axis is linear for the first 0.5 ps followed by a logarithmic scaling from 0.5 to 100 ps. Arrows in the map are aids to follow the trends of continuous spectral shifting referred to in the text. (b) ESA and SE decay of *all-trans* and 13-*cis* AntR. SE at 930 nm from both data sets is multiplied by 2 for clarity.

Estimates of isomerization yields and absorption spectra for both resting states were derived from results presented in Figures 1–4. Within our margin of error in absorption measurement we assumed the alkaline AntR consists exclusively of *all-trans*-retinal pigments.^24^ Next, spectra of the pH 8 AntR sample were measured before and after the imine bond hydrolysis (Figure S3) from which the extinction spectrum of ATAntR is extracted and presented in Figure 5a. Using the well-known correlation of the absorption spectrum of retinal proteins with their C=C bond stretching frequency,^26,27^ a Raman band centered at 1538 cm^–1^ for the 13-*cis* resting state predicts an electronic absorption peak at 525 nm (Figure 5b). We note that C=C stretching in ATAntR is 1531 cm^–1^, predicting an absorption centered at 555 nm and perfectly matching the spectrum under alkaline conditions. Armed with the knowledge of the one spectrum and the peak position of the second, measurements of the difference spectrum between them (see the Supporting Information) leads to an extinction spectrum of 13CAntR as well (Figure 5a).

**Figure 5:**
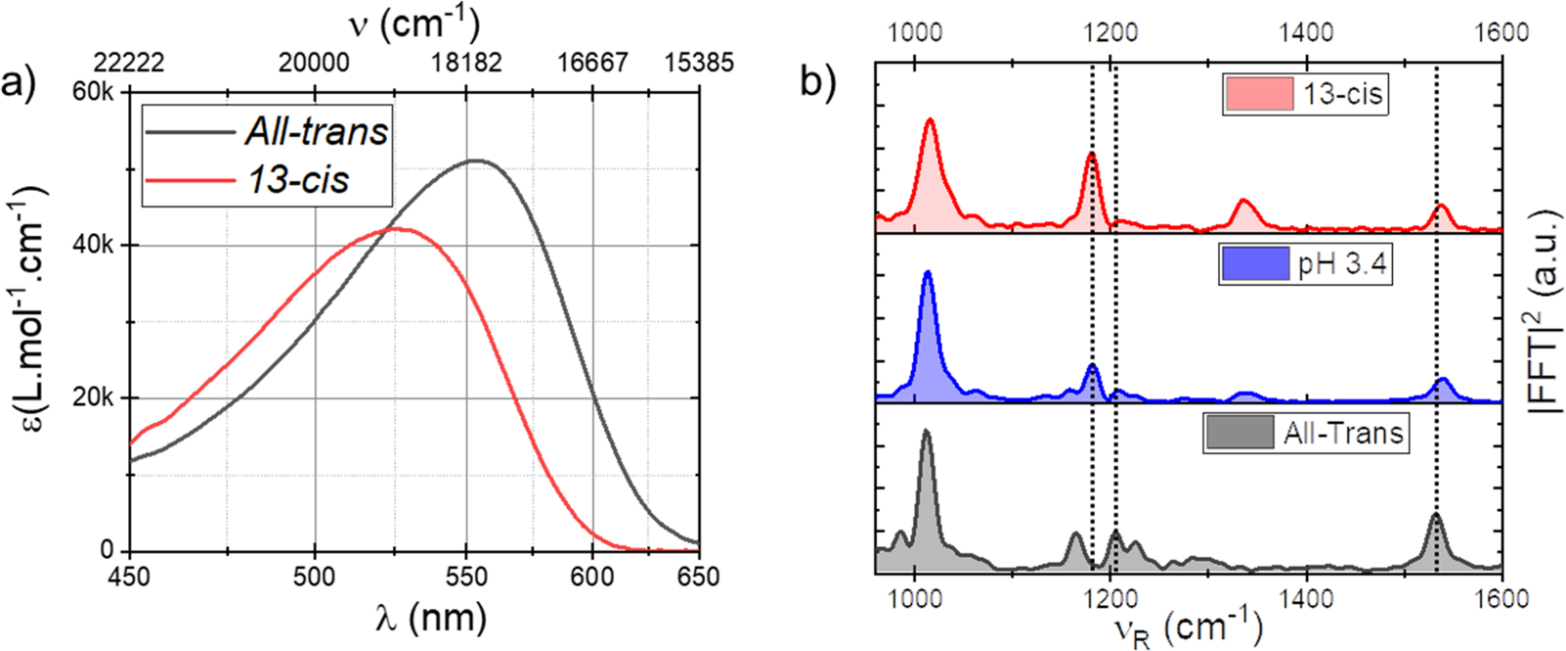
(a) Absorption spectra of *all-trans* and 13-*cis* AntR. (b) Impulsive Raman spectra of pH 8 (*all-trans*), pH 3.4 (*all-trans* and 13-*cis* mixture) and pure 13-*cis*.

The spectra in Figure 5a and comparison of two and three pulse pump–probe experiments on alkaline AntR provide an estimate of ϕ_*at*_, the *all-trans* to 13-*cis* isomerization quantum efficiency. Weak pump–supercontinuum probe sequences were conducted with the former tuned to the K intermediate difference spectrum’s isosbestic point, once with and then without an actinic preparation pulse for generation of a metastable “K” population (see Figure S5). In three pulse experiments, the pump–probe sequence comes 60 ps after the actinic pulse, allowing relaxation either to K or back to ATAntR. Two measures were used to detect the fraction of actinically excited proteins which isomerize. One is the impulsive vibrational signal induced in S_1_ by the pump, and the other is the amplitude of stimulated emission existing at *t* ≥ 1 ps in the pump–probe measurement. Both signals are selective for the remaining ground-state ATAntR when the pump-pulse is incident. After the VV and VVV polarization states of two and three pulse experiments are properly accounted for, the fractional reduction of both measures indicated ϕ_*at*_ = 0.85 ± 0.15. Finally, because of the mixed isomeric composition at low pH, photoisomerization quantum efficiency (ϕ_*cis*_) of 13-*cis* to *all-trans 15-syn* (K′) required comparison of dipole strength ratios between *all-trans* and 13-*cis* resting-states to be used for estimating ϕ_*cis*_ = 0.5 ± 0.15 (for details, see the Supporting Information).

Before considering the mechanistic basis for photodynamic similarities in these three proteins, we review assumptions employed in our analysis. Unlike BR and ASR, shifting resting-state equilibrium in AntR is achieved by changes in pH, which could involve two protomers and/or two retinal configurations. Here we have chosen the simplest scenario consistent with our observations, whereby the *all-trans* configuration is identical at both acidities. This matches the identical excited-state spectra and isomerization constants of their fluorescent state, and the same CC stretching frequencies observed in Raman. Ballistic photoisomerization should be enabled by several characteristics of the potential surface topology. First, strong forces must act in the Franck–Condon (FC) state directing motion to the crossing seam. Implicit in this statement is the absence of any barriers requiring thermal activation or energy redistribution to go between these two crucial regions of phase space, possibly arising from state mixing between excited singlet surfaces.^28^ Why a 13-*cis* configuration ensures this in MRPs requires explanation. Another aspect of electronic structure which could further facilitate such dynamics is structural similarity between the FC state and the crossing region requiring less structural reorganization and energy redistribution. Such similarity in structure between the resting state and the crossing seam is unlikely since it would predict low quantum yields for isomerization contrary to observations.^29,30^ The question of pretwisting effects on reaction rates has been discussed extensively in the context of photoisomerization in bovine rhodopsin.^31−33^ Some studies have suggested that the 11-*cis* isomer of the protonated retinal Schiff base, at least in one of its stable conformations, is even in solution prone to direct wave packet-like internal conversion.^34^ Others have suggested that structural interactions with the protein surroundings impose prestraining, providing impetus for such dynamics.^35,36^ However, these factors may not translate from 11- to 13-*cis* isomers of this molecule when interactions with the protein are included. Activity of HOOP vibrations in resonance Raman spectra have been taken as markers for pretwisting in retinal proteins. While no such information exists for AntR, BR and ASR do show strong HOOP activity, which is specific for the 13-*cis* isomers, consistent with the pre-existing deformation playing a role in the different reaction dynamics.^37,38^

Other structural characteristics of 13-*cis* resting state which could impact the dynamics of photoisomerization are related to the disruption of the hydrogen-bonded networks surrounding the Schiff base due to transformation from a 15-*syn* to a 15-*anti* configuration. Steric conflicts between hydrogens near the anchoring lysine residue have often been suggested to enhance shifts from planarity of the polyene in its DA state.^39^ Deviations from planarity of the retinal chromophore in its 13-*cis* ground state were detected for both BR and ASR. Prestraining for the 13-*cis* isomer was found in BR not only by Raman methods^37^ but also in NMR studies in which an unusual ^13^C chemical shift was detected for C_14_ pointing to C_14_–C_15_ twist.^40^ In addition, X-ray data of BR indicated a twist around the C_14_–C_15_ bond in the 13-*cis* retinal isomer in contrast to the *all-trans* isomer.^41^ Furthermore, the blue-shifted absorption of the 13-*cis* isomer of BR, ASR, and AntR supports a twisted conformation with disrupted bond conjugation. Thus, the twist around the C_14_—C_15_ bond, which is adjacent to the active C_13_=C_14_, may play a role in the faster dynamics of 13-*cis* retinal. A recent femtosecond X-ray study of BR has depicted the importance of the specific electrostatic interactions between the protein and the retinal protonated Schiff base to guide the isomerization in a certain direction.^42^ Different electrostatic retinal–protein interactions of the *all-trans* and 13-*cis* isomers may lead to different dynamics during the isomerization reaction. FTIR study of BR showed that the retinal isomerization caused a stronger disruption of the water–protonated Schiff base hydrogen bond in the *all-trans* as compared to 13-*cis* state, which may contribute to the faster isomerization dynamic of the 13-*cis* isomer as well.^43^

The low-temperature FTIR studies of the primary photointermediates (the K states) of microbial rhodopsins also point to similarities between the mechanisms of retinal photoisomerization in 13-*cis* states of BR, ASR, and AntR and their difference from the respective *all-trans* states. Specifically, all the K intermediates derived from the 13-*cis* (but not *all-trans*) states show extensive HOOP bands, which may be explained by the fact that bond twists are concentrated in the Schiff base area for *all-trans* pigments and spread along the polyene chain for 13-*cis* pigments.^43,44^ These common structural aspects of the 13-*cis* states of the three proteins may thus contribute to the exceptionally rapid photoisomerization in them all. Further establishing generality of this trend will require testing of additional proteins which accommodate stable 13-*cis* 15-*syn* resting states, and its mechanistic deciphering necessitates comparative modeling of them all with theory. It is noteworthy that the case of AntR is unique in that the method of stabilizing the *cis* configuration is also thought to involve counterion protonation, a factor which could have specific equally unique consequences on reactivity.

Nonetheless, the bidirectional photoisomerization dynamics measured in AntR possibly marks the discovery of a first robust trend in photochemical reactivity of microbial rhodopsins which have so far proven elusive. Finally, torsional excited-state coherences observed in TA are used here to determine isomerization efficiency in mixed isomer states, demonstrating the utility of these observables beyond the extraction of reaction dynamics. Key to this application are high time resolution, low noise levels, and broad band dispersed detection of the collected signals.

## Experimental Methods

AntR sample preparation was similar to that described in ref (24). TA measurements were performed as detailed elsewhere.^45^ Slight modifications of sample preparation and in TA data collection are fully described in the Supporting Information.

## Supporting Information Available

Experimental method, temporal characterization of pump, SE decay of *all-trans* AntR and its fitting, impulsive vibration, absorption spectra of retinal isomers, photochemistry of K intermediate and quantum efficiency of *all-trans 15-anti* to K isomerization, and isomerization quantum efficiency of *13-cis 15-syn* to *all-trans 15-syn* (PDF)
