# Supplementary material for: Bidirectional Photochemistry of Antarctic Microbial Rhodopsin: Emerging Trend of Ballistic Photoisomerization from the 13-cis Resting State
Source: J Phys Chem Lett. 2022 Aug 24;13(34):8134–40. doi: 10.1021/acs.jpclett.2c01974 (PMC9442786; doi:10.1021/acs.jpclett.2c01974)
Supplement: Supplementary file 1 — jz2c01974_si_001.pdf [file jz2c01974_si_001.pdf]

Supporting Information

Bidirectional Photochemistry of Antarctic Microbial Rhodopsin: Emerging Trend of Ballistic Photo-isomerization from the *13-cis* Resting State

Partha Malakar[a], Ishita Das[b], Sudeshna Bhattacharya[b], Andrew Harris[c], Mordechai Sheves[b], Leonid S. Brown[c], Sanford Ruhman*[a]

[a]Institute of Chemistry, The Hebrew University of Jerusalem, Jerusalem, 9190401, Israel.

[b]Department of Molecular Chemistry and Materials Science, The Weizmann Institute of Science, Rehovot 7610001, Israel

[c]University of Guelph, Department of Physics, 50 Stone Rd. E., Guelph, Ontario N1G 2W1, Canada

<u>Table of contents:</u>

Experimental methods	p2-p3
Temporal characterization of pump 1	p4
SE decay of *all-trans* AntR and its fitting	p5
Impulsive vibrations	p6
Absorption spectra of retinal isomers	p7-p9
Photochemistry of K intermediate and quantum efficiency of *all-trans 15-anti* to K isomerization	p9-p15
Isomerization quantum efficiency of *13-cis 15-syn* to *all-trans 15-syn*	p15-p17
References	p18

Experimental Methods:

AntR expression and purification: The gene encoding AntR (Ga0105045_102227662) were cloned into pET21a(+) vector by GenScript, with a C-terminal 6×His-tag, as previously reported.[S1] Transformation of the *Escherichia coli* C41 (DE3) strains with the plasmids was done by Lucigen's heat-shock transformation protocol.[S1] The cells were grown overnight at 130 rpm in 5 mL 2×YT media (1% yeast extract, 1.6% tryptone, 1% NaCl) with 100 µg/ml ampicillin at 37°C. 2 mL of the cell culture was used to inoculate 1 L of 2×YT media in presence of 100 µg/ml ampicillin (at 37°C, 130 rpm), and grown to an optical density OD_{600} of 0.6. Overexpression was induced by 1 mM isopropyl-β-D-thiogalactoside (IPTG), followed by addition of 10 µM all-*trans* retinal, and additional shaking for 8 hrs. The cells were then collected by centrifugation at 7000 rpm and 4 °C for 15 min. The collected cell pellet was re-suspended in 50 mM MeS [2-(N-morpholino)-ethanesulfonic acid] buffer pH 6, 300 mM NaCl, 5 mM imidazole and 5 mM $MgCl_2$.The suspension was lysed with 0.2 mg/mL lysozyme overnight at 4°C in the presence of 2 µg/mL DNase and 1.5% (w/v) DDM (n-dodecyl-β-D-maltoside). DDM solubilized protein was separated as supernatant by centrifugation at 18000 rpm and 4°C for 35 min, and then loaded on Co^{2+}-NTA resin (ThermoFisher scientific) pre-equilibrated with 50 mM MES buffer containing 300 mM NaCl, 5 mM Imidazole at pH 6. Other protein contaminants were removed by washing with 50 mM MES buffer containing 300 mM NaCl, 50 mM Imidazole, and 0.06% DDM, at pH 6. Protein elution was done with an elution buffer (50 mM Tris-HCl, 300 mM NaCl, 200 mM Imidazole, 0.06% DDM; pH 7.5). The desired purified protein was obtained followed by washing off the imidazole by using an Amicon Ultra centrifugal filter tube (10kDa MWCO) by three times repeated solvent exchange with 0.02% DDM and 100 mM NaCl. The concentrated AntR was stored in 0.06% DDM with 100 mM NaCl for further use. For the spectroscopic measurements,

AntR was dissolved in Tris buffer at pH 8 with 0.06% DDM, and 100 mM NaCl or Sodium acetate buffer of pH 3.4 (with 0.06% DDM, 100 mM NaCl).

TA measurements: TA measurements were performed using a homemade flow cell with a 0.25 mm path length equipped with 0.15 mm glass windows. A syringe pump is employed for flowing, and its speed is tuned so that consecutive laser pulses excite fresh AntR, and signals are not dependent on increase of flow rate. Sample integrity is monitored consistently by taking absorption spectra throughout the measurement. A hybrid multipass amplified Ti-sapphire laser system that generates 30 fs, 1 mJ pulses at ~800 nm with a 1000 Hz repetition rate was used for TA measurement. Details of the detection set-up and supercontinuum probe generation are detailed elsewhere.[S2] The pump pulses are generated on a home-built noncollinear optical parametric amplifier (NOPA), generating tunable broad-band pulses between 500-700 nm wavelength. The NOPA output is compressed using a pair of chirp mirrors (LASER QUANTUM, UK) and a 19-channel deformable mirror coupled to a grating compressor.[S3] The temporal profile of NOPA output is characterized by Frequency gated optical grating method (SI figure S1). Probing in the NIR is performed using the same laser set-up but at a reduced 370 Hz repetition rate. The supercontinuum probe pulses are generated using a fraction of 800 nm fundamental output on a 3 mm sapphire crystal. The dispersed probe pulses are detected on an InGaAs NIR sensor (B&W TEK, USA). Otherwise, set-up is as used for the visible measurements.

Temporal characterization of pump 1:

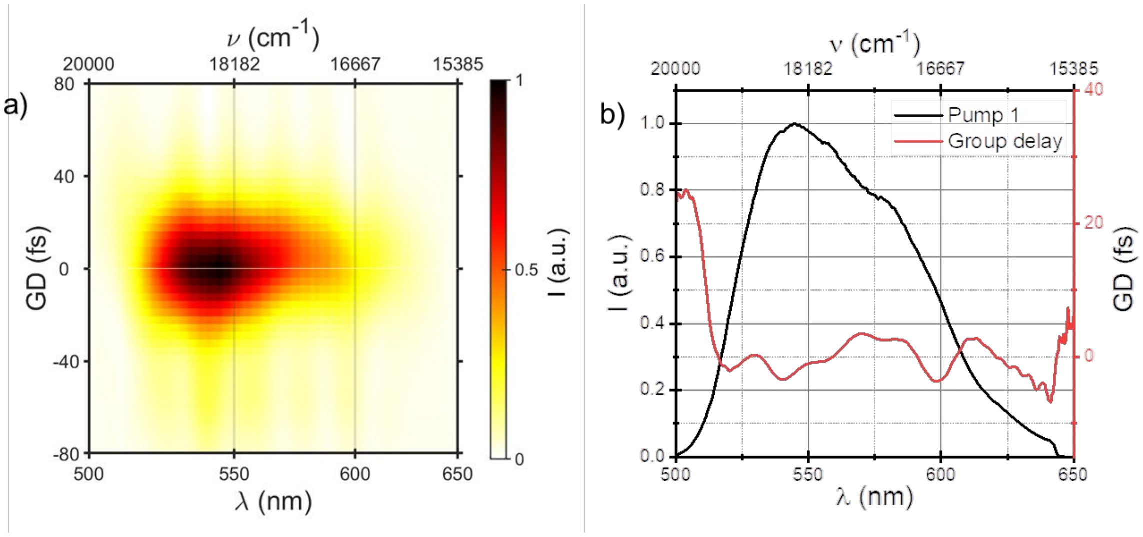

Figure S1: (a) 2D colour map of FROG for the pump 1. X-axis represents wavelength, the y-axis represents the group delay, and transmitted intensity is colour coded; (b) The pump 1 spectrum and group delay obtained from FROG. The left y-axis (black) stands for normalized pump intensity, and the right y-axis (red) represents group delay.

<u>SE decay of *all-trans* AntR and its fitting:</u>

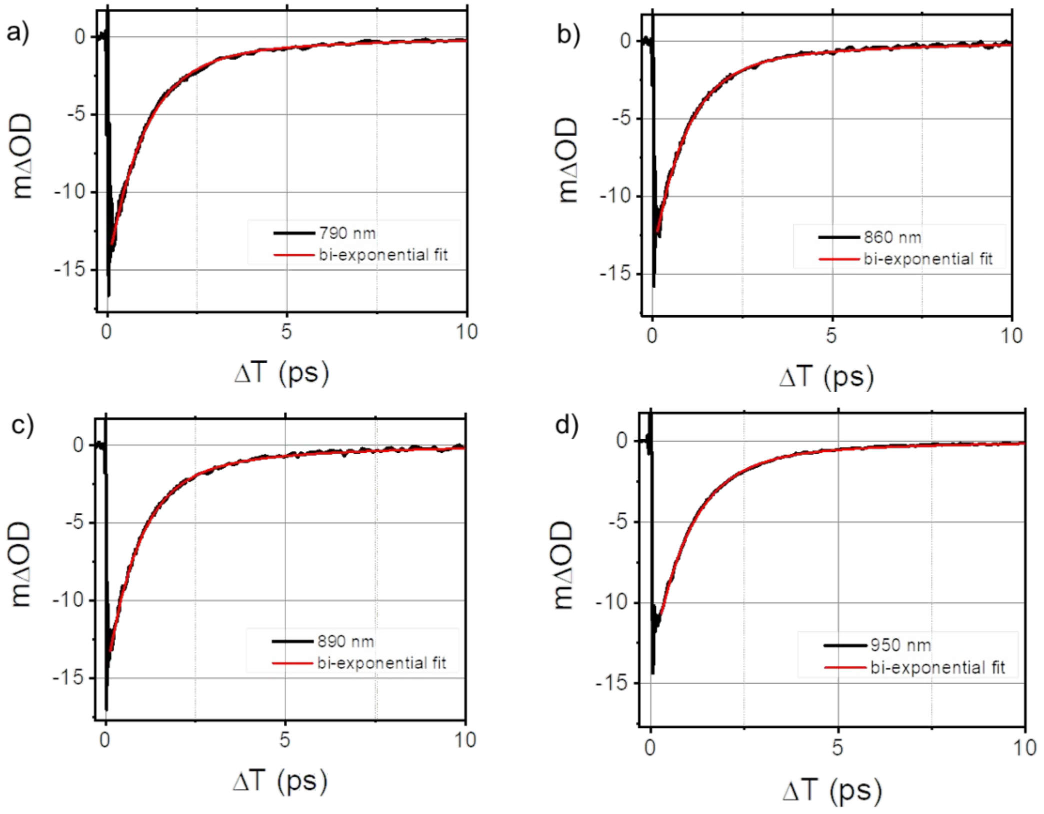

Figure S2: Stimulated emission decay and biexponential [$\Delta OD(t) = A_1 exp(-t/\tau_1) + A_2 exp(-t/\tau_2)$] fitting at various probe wavelength for the pH 8 sample upon excitation with pump 1. The fitting parameters are presented in table 1.

Table 1: Fitting results such as lifetime, amplitude, and average lifetime at various probe wavelengths

λ_p (nm)	τ_1 (fs, ±50)	A_1(mΔOD, ± 0.5)	τ_2 (fs, ±500)	A_2 (mΔOD, ±0.5)	τ_{av} (fs, ±100)
790	1000	14 (93%)	5500	1 (7%)	1320
860	900	12 (86 %)	5000	2 (14 %)	1470
890	900	13 (86 %)	4300	2 (14 %)	1340
950	1000	13 (93 %)	4900	1 (7 %)	1270

Impulsive vibration:

Excitation with ultrashort pulse generates coherent wavepacket motion in the ground and excited state, which is detected in the TA data as a periodic modulation at the early delay.[S4] Periodic modulations are separated by the biexponential fitting of individual wavelength kinetics data. Fourier transform of such time-domain modulation provides the frequency of the oscillations. Fourier analysis of pH 8 (From the data presented in figure 2a) shows peaks assignable to ethylenic stretching at 1531 cm^{-1}, to C-C stretching motions at 1220, 1200, 1160 and in-plane methyl rocking at 1010 cm^{-1} are identical to reported frequency domain spontaneous Raman measurements (Figure 5b).[S1] In addition, impulsive peaks not observed in spontaneous Raman are detected at 900, 840, 780, 205 and 170 cm^{-1}. Such peaks are probably due to the Raman activity of the excited state.[S5] These features will be addressed in detail elsewhere. On the other hand, a similar analysis of pH 3.4 (from data presented in figure 2c) shows additional, prominent peaks at 1180 and 1340 cm^{-1} (Figure 5b). Characteristic *all-trans* peaks 1220, 1220 and 1160 remained, but the Fourier intensity was reduced. In addition, ethylenic stretch shifted to 1537 cm^{-1}. The peaks for pH 3.4 are in good agreement with the reported spontaneous Raman.[S1] Fourier analysis of pure *13-cis* (From the data presented in figure 4a) shows ethylenic stretch at 1538, C-C stretch at 1180 and in-plane methyl rock at 1010 cm^{-1} (Figure 5b). Interesting to note that the C-C stretch assignable to *all-trans* remained unaffected by the pH change of the medium. Such invariance suggests the environment around the *all-trans* retinal protonated Schiff base remained unaffected by the acidification.

Absorption spectra of retinal isomers:

A dilute sample of AntR ($OD_{555} \approx 0.085$) was made to react with 400 mM hydroxylamine solution at pH 8 for 15 min, under light irradiation with a Schott 250 W cold light source (with a long-pass >520 nm) cut-off filter. The reaction with hydroxylamine produced retinal oxime characterized by a blue-shifted absorption band at ~365 nm (figure S3a). From a difference absorption spectrum obtained between before and after the hydroxylamine reaction, as shown in figure S3b, the ratio of ΔOD_{555} (for retinal pigment) and ΔOD_{365} (for retinal oxime) was estimated. Using the molar extinction coefficient of retinal oxime[S6] as 33600 M^{-1} cm^{-1}, and the value of $\Delta OD_{555}/\Delta OD_{365}$, the extinction coefficient of AntR was determined as 51000 ± 1000 M^{-1} cm^{-1}.

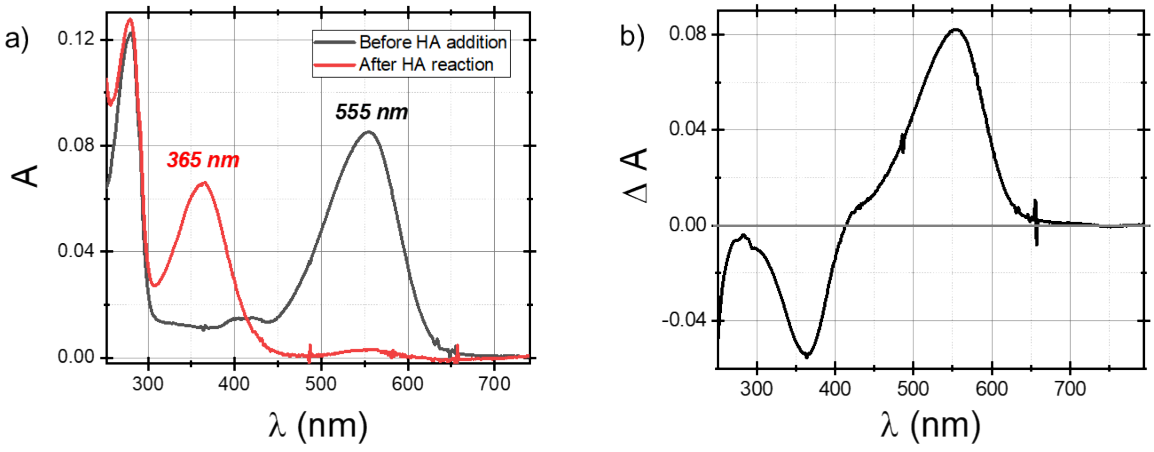

Figure S3: (a) Absorption spectra of AntR before and after hydroxylamine reaction. (b) Corresponding difference absorption spectrum.

AntR sample at pH 3.4 contains both isomers, but at pH 8 almost exclusively, the *all-trans* isomer is present. Therefore, the *13-cis* AntR absorption spectrum could be isolated if its absorption maximum and difference with the *all-trans* state are known. We evaluated the absorption maximum of *13-cis* state from C=C stretch and absorption maximum frequency

correlation.[S7] Pure 13-*cis* AntR exhibits C=C stretching at 1538 cm^{-1}, predicting an absorption maximum at 525 nm. On the other hand, light-dependent bi-stability at pH 6 is assigned to the isomeric composition variation. Under blue light (< 480 nm) illumination, the absorption maximum is located at 547 nm, but as the light turned to red (> 560 nm), the maximum shifted to 542 nm. Such a redshift in the absorption maximum is assigned to the increased all-*trans* fraction.[S1] Hence, the absorption difference between blue and red (blue - red) light irradiation represents *all-trans* minus *13-cis* state spectrum (Figure S4a). Therefore, using prior knowledge of absorption maximum absorption difference with *all-trans,* we evaluated *13-cis* state absorbance (figure 5a main text).

Knowing the individual isomer's absorbance, we reconstructed the pH 3.4 absorbance (figure S4 b). An acidic sample contains about 60 ± 5% *13-cis* and 40 ± 5 % *all-trans* retinal. Figure S4 b

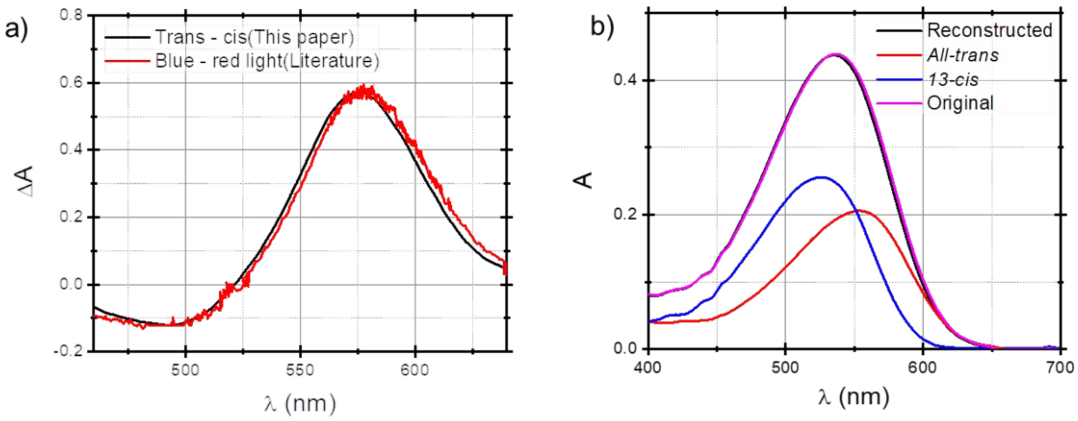

shows the identical absorbance of individual isomers at the pump wavelength maximum. Therefore, pump 1 excites individual isomers to the same extent. As a result, the finite difference (discussed in the main text and figure 3) estimated a 35 ± 5 % abundance of *all-trans* retinal in the pH 3.4. The absorption spectrum of the *13-cis* state (figure s4 b) will be used for determining its photoisomerization quantum yield later.

Figure S4: (a) Difference spectra between the *all-trans* and *13-cis* states; (b) Absorption spectra of pH 3.4 AntR along with its *all-trans* and *13-cis* components.

Photochemistry of the K intermediate and quantum efficiency of *all-trans 15-anti* to K isomerization:

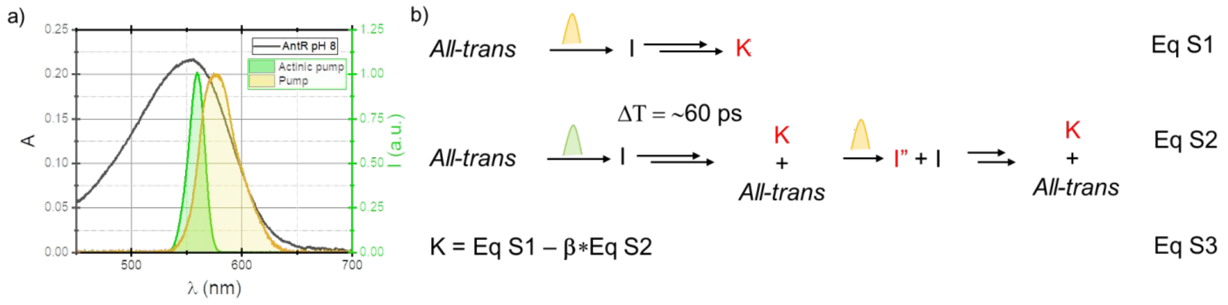

Figure S5: (a) Absorption and excitation pulse spectra; (b) Isolation of the K intermediate’s photochemistry. Probe and pumps have identical electric field polarization.

Photoexcited *all-trans* AntR either isomerizes to *13-cis* J intermediate or returns to *all-trans* S0 state. J intermediate later cools to K intermediate within a few picoseconds, which lives for a microsecond. Therefore, after 60 ps from the start of the photocycle, TA will record both *all-trans* state and K. On the other hand, before commencing the photocycle, TA will record only the *all-trans* state. Knowing the *all-trans* components, pure K information could be isolated. The *all-trans* absorption spectrum and excitation pulse spectra are shown in figure S5a. Pulse sequence and extraction of individual component information are presented in figure S5b.

We used an ~ 100 fs actinic pulse with a wavelength centred at 560 nm to excite ~12 ± 2 % of the *all-trans* ground state (Table S2). Next, after 60 ps of the actinic excitation, we measured the pump-probe on the mixture. Hence the resultant TA data (VVV) consist of both K and *all-trans* information (figure S6a). Following photoexcitation, the TA spectrum shows characteristic excited state absorption, ground state bleach, and stimulated emission. However, excited-state absorption and stimulated emission show a fast-decaying component over the first 300 fs and a picosecond component. On the other hand, TA measurement in the absence of actinic excitation (VV) shows only the picosecond component. Therefore, the fluorescent state of K intermediate decay much

faster than that of the *all-trans* state. Again, this is similar to pH 3.4, where *13-cis* form reacting faster than *all-trans*. Therefore, the all-trans component in the VVV data is reduced to 78 ± 3 % of VV using dynamic difference spectra.

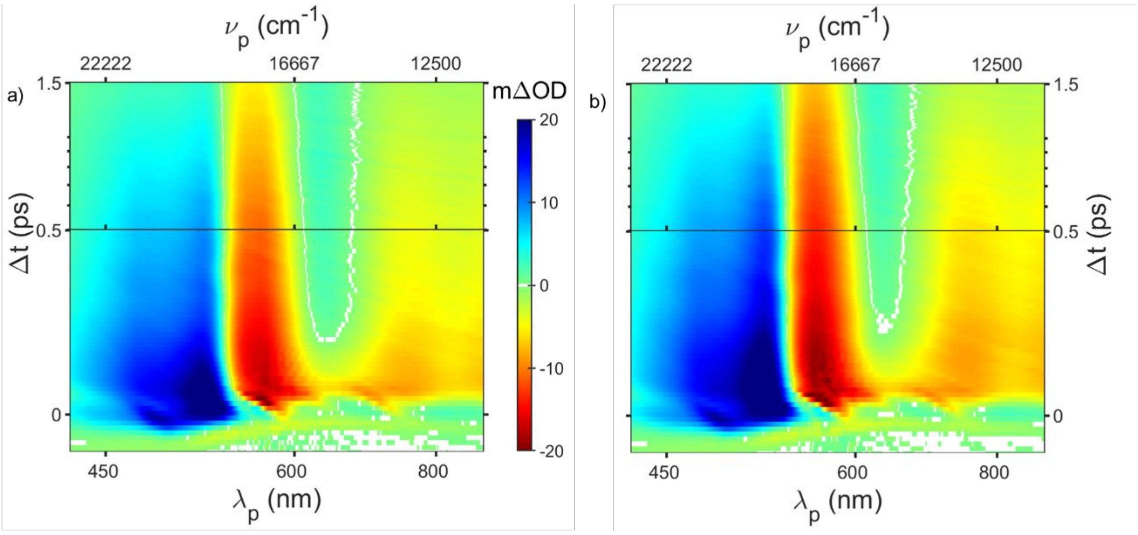

Figure S6: (a) 2D colour map of TA data (VVV) of dark-adapted AntR at pH 8 in the presence of actinic excitation as described in Eq S2. The X and Y axes represent probe wavelength (λ_p) / wavenumber (ν_p) and delay between pump and probe (Δt), respectively. The time axis is linear for the first 0.5 ps, followed by a logarithmic scale from 0.5 -1.5 ps. ΔOD colour-coding is depicted in the attached scale; (b) 2D colour map of TA data (VV) of dark-adapted AntR at pH 8 in the absence of actinic excitation as described in Eq S1. All axes of (b) are the same as (a).

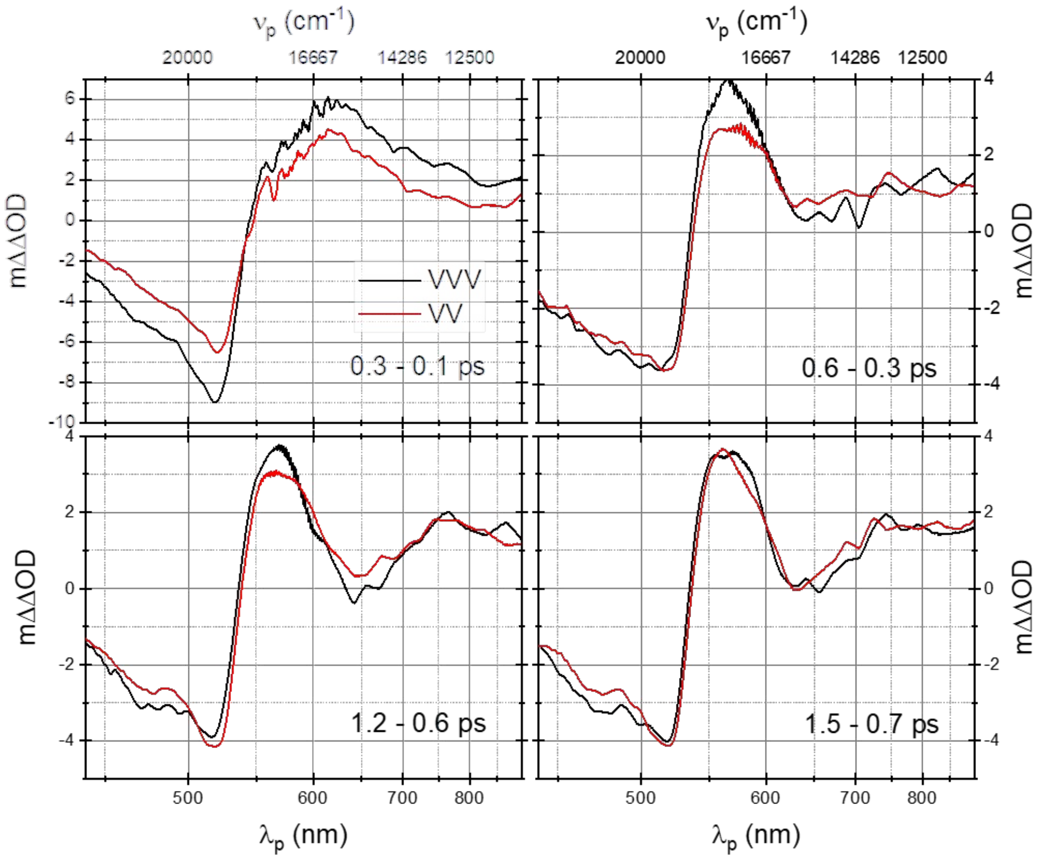

Figure S7: Dynamic difference spectra [ΔOD(t+δt)-ΔOD(t)], at various stages of excited state decay. The Black and red lines represent dynamic difference spectra from VVV and VV set, respectively. VV spectra are multiplied by 0.78. The relevant time information is provided in each graph.

On the other hand, VV data shows pronounced vibrionic modulation near 530 nm probe wavelength due to Raman vibrations of the *all-trans* manifold. However, the amplitude of such modulation is reduced for VVV data set. Interestingly, a comparison of vibronic modulation also yields an identical reduction of *all-trans* components from VV to VVV. Therefore, the Raman vibration of the *all-trans* manifold could be used as a quantitative marker.

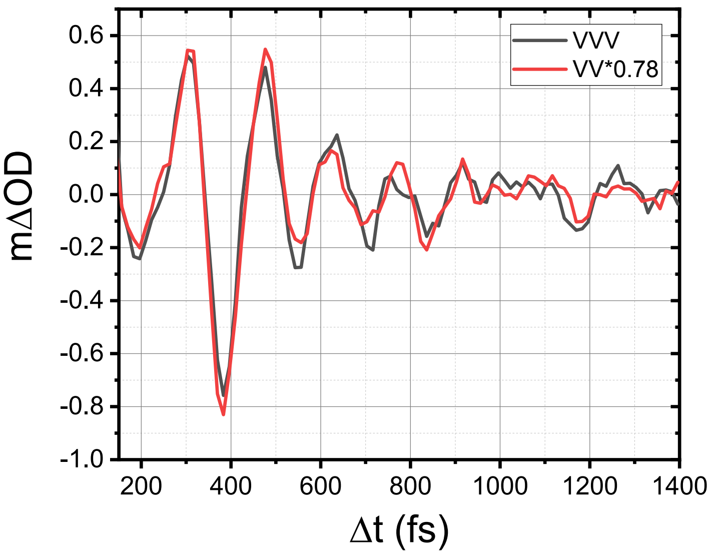

Figure S8: Vibronic modulation at 530 nm in presence (VVV) and absence (VV) of the actinic excitation.

The retinal's transition dipole is positioned along the linear retinal backbone, causing TA anisotropy of 0.4, identical to the theoretical estimate. Therefore, we need to consider the effect of the photo selection because of the specially oriented sample.[S8] The primary pump excites a homogeneous population. We are probing with an identical polarization of light after the excitation with a linearly polarised light need $\cos^2\theta$ special integration. Similarly, a three-pulse measurement consisting of excitation, re-excitation and probing with the same electric field polarization requires $\cos^4\theta$ special integration.

We assume the following:

1) Only the actinic pump will include depletion effects

2) Even in the actinic interaction, the depletion will only be introduced by an average intensity assumed uniform in sample depth.

Table S2: The excitation parameters

Photon density (J) m^2	Absorption cross-section at 560 nm (σ) m^{-2}	% excited at the magic angle	% excited at the identical pump and probe polarization
$(6.4 \pm 0.5) \times 10^{18}$	$(1.9 \pm 0.1) \times 10^{-20}$	12 ± 2	22 ± 4

% excitation at magic angle = 100*e$^{(-J\sigma)}$;

% excitation at identical pump and probe polarization = % excitation at magic angle×(9/5);

Our pump-probe stage is the basis for interrogation with a comparison with and without the actinic interaction. We know that the actinic pump-probe leads to a 22 ± 4% S_0 bleach just after the pump.

So:

$$\frac{\int_0^{2\pi}\int_0^{\pi} e^{-Acos^2\theta} cos^2\theta \; sin\,\theta \; d\theta \; d\varphi}{\int_0^{2\pi}\int_0^{\pi} cos^2\theta \; sin\,\theta \; d\theta \; d\varphi} = 0.78$$

Or

$$3\left(\frac{1}{3} - \frac{A}{5} + \frac{A^2}{14} + ..\right) = 0.78$$

Or A = 0.43 ± 0.7

The cos^2 factor relates to the probing interaction, with the integral in the denominator representing the probe signal without actinic depletion. By running these integrals with various values of A we can find the correct factor representing $J\sigma$ and assuming unity quantum efficiency. A value of A=0.43 provided the right depletion measure.

Giving a ratio of 0.78. This factor can then be used to assess the effect of this same depletion on the signal in a secondary pump-probe sequence: $\frac{\int_0^{2\pi}\int_0^{\pi} e^{-Acos^2\theta} cos^4\theta \, sin\,\theta \, d\theta \, d\varphi}{\int_0^{2\pi}\int_0^{\pi} cos^4\theta \, sin\,\theta \, d\theta \, d\varphi}$. This results in the following value:

$$\frac{\int_0^{2\pi}\int_0^{\pi} e^{-0.43cos^2\theta} cos^4\theta \, sin\,\theta \, d\theta \, d\varphi}{\int_0^{2\pi}\int_0^{\pi} cos^4\theta \, sin\,\theta \, d\theta \, d\varphi} = 0.74 \pm 0.04$$

and so $\frac{\int_0^{2\pi}\int_0^{\pi} e^{-Acos^2\theta} cos^4\theta \, sin\,\theta \, d\theta \, d\varphi}{\int_0^{2\pi}\int_0^{\pi} cos^4\theta \, sin\,\theta \, d\theta \, d\varphi} = 0.74 \pm 0.04$ providing an estimated bleach in the pump-probe signal of 26 ± 4 % if the quantum efficiency is 100 %.

Dynamic difference or vibronic modulation measure shows 22 ± 3 % reduction of all-*trans* state concentration following actinic excitation. Therefore, isomerization efficiency is 85 ± 15 %.

Isomerization quantum efficiency of *13-cis 15-syn* to *all-trans 15-syn*:

Experimentally, isomerization quantum yield determination of the *13-cis* resting-state remained challenging due to the absence of a pure *13-cis* sample. In general, the ground state *all-trans* isomer of MRP has red-shifted absorption and higher absorbance than its 13-*cis* isomer. Therefore, all-*trans* state has a higher transition dipole than is *13-cis* isomer. Since transition dipole is proportional to the integrated extinction coefficient (Σ), the Σ ratio between *all-trans* and *13-cis* states will provide the dipole strength ratio. Knowing the individual absorption spectra, calculated transition dipole ratio between *all-trans* and *13-cis* states is 1.18 : 1 of ground state AntR (figure S9). Performing similar analysis on the absorption spectra of other MRP's like BR and ASR provides identical dipole strength ratio.[S9,S10]

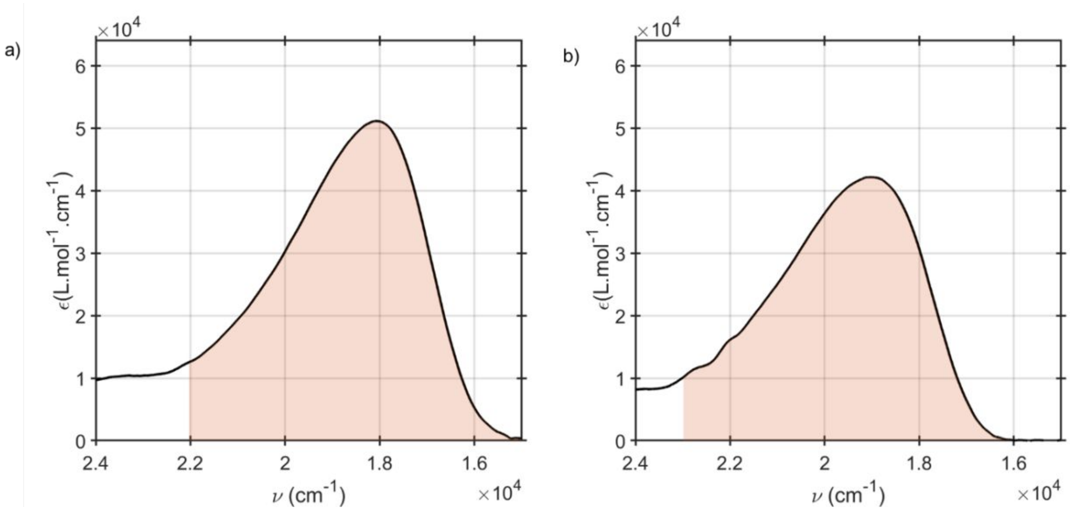

Figure S9: (a) Abortion spectrum of *all-trans* AntR (black line) and the highlighted area is integrated. Integration from the start of the absorption (15400 cm^{-1}) to 22000 cm^{-1} provides a value of 1.9 x 10^8 $L.mol^{-1}.cm^{-2}$. (b) The absorption spectrum of *13-cis* AntR and its integration. In order to make a viable comparison same 6600 cm^{-1} width from the start of the absorption is integrated. Integration of *13-cis* state absorption is 1.6 x 10^8 $L.mol^{-1}.cm^{-2}$.

Photoexcited *13-cis 15-syn* resting-state isomerized to *all-trans 15-syn* (K') ground state within sub 100 fs. The TA spectra of *13-cis* isomer at a longer Δt show a more positive ΔOD portion than its negative part, indicating transition dipole increases following isomerization. Hence, the K' spectrum is red-shifted with an increased absorbance like an *all-trans* resting state. We assume that the transition dipole ratio between *all-trans* and *13-cis* is invariant, and its value remains 1.18 : 1. According to our assumption, the transition dipole ratio between K' and *13-cis* must be 1.18 : 1.

To calculate the quantum efficiency of isomerization, number of excited chromophores and the number of those isomerized are required. Knowing the density of excitation and absorption, cross-section (table S3) excitation fraction is calculated and absorption is presented in figure s10 a. Next,

from the integrated area of the TA spectra at 200 ps, assuming transition dipole ratio of *all-trans* and *13-cis* isomer, reacted *13-cis* absorbance is presented in figure s10 b. Comparing these two absorption spectra yields a quantum efficiency of 50 ± 15 %.

Table S3: The excitation parameters

Photon density (J) m^2	Absorption cross-section at 545 nm (σ) m^{-2}	% excited at the magic angle	% excited at the identical pump and probe polarization
(4.5 ±0.5) × 10^{18}	(1.3 ±0.1) × 10^{-20}	5 ± 1	9 ± 2

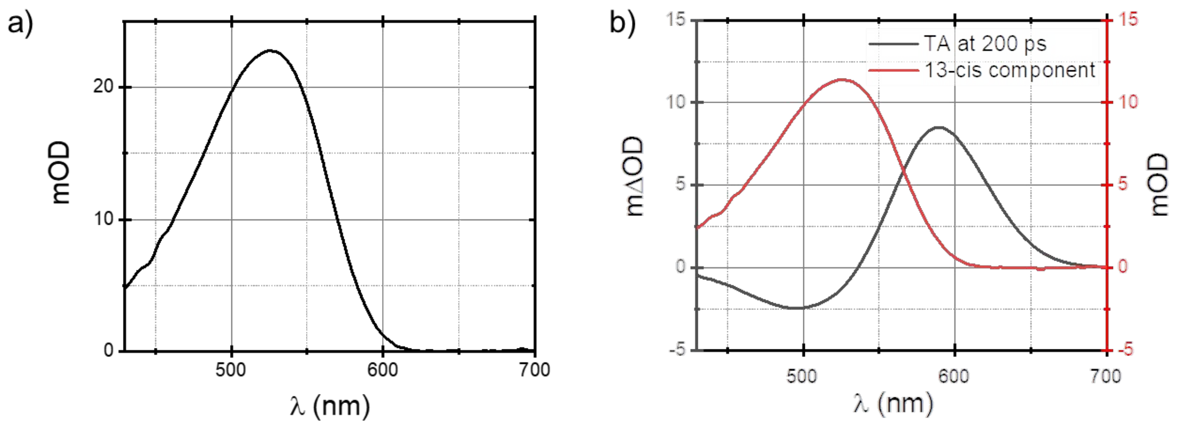

Figure S10: (a) *13-cis* state absorbance calculated from absorption cross-section and photon density; (b) TA spectra of *13-cis* state at 200 ps (black) and reacted *13-cis* state (red) absorption. Since the TA spectra at Δt = 200 ps is integrated from 14000 to 23000 cm^{-1}, ratio of areas between *all-trans* and *13-cis* states spectra to be used for 13-*cis* absorbance separation. Integrated area ratio between *all-trans* and *13-cis* states for such range is 1.23. Next, using the integrated area of TA difference spectra at 200 ps and integrated area ratio between *all-trans* and *13-cis* states of 1.23, reacted *13-cis* absorbance is isolated.

References:

[S1] Harris, A.; Lazaratos, M.; Siemers, M.; Watt, E.; Hoang, A.; Tomida, S.; Schubert, L.; Saita, M.; Heberle, J.; Furutani, Y.; Kandori, H.; Bondar, A.-N.; Brown, L. S. Mechanism of Inward Proton Transport in an Antarctic Microbial Rhodopsin. *J. Phys. Chem. B* **2020**, *124* (24),4851-4872.

[S2] Dana, J.; Haggag, O. S.; Dehnel, J.; Mor, M.; Lifshitz, E.; Ruhman, S. Testing the Fate of Nascent Holes in CdSe Nanocrystals with Sub-10 Fs Pump–Probe Spectroscopy. Nanoscale 2021, 13 (3), 1982–1987.

[S3] Kahan, A.; Nahmias, O.; Friedman, N.; Sheves, M.; Ruhman, S. Following Photoinduced Dynamics in Bacteriorhodopsin with 7-Fs Impulsive Vibrational Spectroscopy. *J. Am. Chem. Soc.* **2007**, *129* (3), 537–546.

[S4] Pollard, W. T.; Dexheimer, S. L.; Wang, Q.; Peteanu, L. A.; Shank, C. V.; Mathies, R. A. Theory of Dynamic Absorption Spectroscopy of Nonstationary States. 4. Application to 12-Fs Resonant Impulsive Raman Spectroscopy of Bacteriorhodopsin. *J. Phys. Chem.* **1992**, *96* (15), 6147–6158.

[S5] Kraack, J. P.; Buckup, T.; Hampp, N.; Motzkus, M. Ground- and Excited-State Vibrational Coherence Dynamics in Bacteriorhodopsin Probed With Degenerate Four-Wave-Mixing Experiments. *ChemPhysChem* **2011**, *12* (10), 1851–1859.

[S6] Iwamoto, M.; Sudo, Y.; Shimono, K.; Kamo, N. Selective Reaction of Hydroxylamine with Chromophore During the Photocycle of Pharaonis Phoborhodopsin. *Biochim. Biophys. Acta* **2001**, 1514, 152-158.

[S7] Kochendoerfer, G. G.; Wang, Z.; Oprian, D. D.; Mathies, R. A. Resonance Raman Examination of the Wavelength Regulation Mechanism in Human Visual Pigments. *Biochemistry* **1997**, *36* (22), 6577–6587.

[S8] Magde, D. Photoselection with Intense Laser Pulses. *J. Chem. Phys.* **1978**, *68* (8), 3717–3733.

[S9] Hofrichter, J.; Henry, E. R.; Lozier, R. H. Photocycles of Bacteriorhodopsin in Light- and Dark-Adapted Purple Membrane Studied by Time-Resolved Absorption Spectroscopy. *Biophysical Journal* **1989**, *56* (4), 693–706.

[S10] Cheminal, A.; Léonard, J.; Kim, S.-Y.; Jung, K.-H.; Kandori, H.; Haacke, S. 100 Fs Photo-Isomerization with Vibrational Coherences but Low Quantum Yield in Anabaena Sensory Rhodopsin. *Phys. Chem. Chem. Phys.* **2015**, *17* (38), 25429–25439.
